# Tumor Cell-Secreted ISG15 Promotes Tumor Cell Migration and Immune Suppression by Inducing the Macrophage M2-Like Phenotype

**DOI:** 10.3389/fimmu.2020.594775

**Published:** 2020-12-23

**Authors:** Ren-Hui Chen, Zhi-Wen Xiao, Xiao-Qing Yan, Ping Han, Fa-Ya Liang, Jing-Yi Wang, Shi-Tong Yu, Ting-Zhen Zhang, Si-Qi Chen, Qian Zhong, Xiao-Ming Huang

**Affiliations:** ^1^ Guangdong Provincial Key Laboratory of Malignant Tumor Epigenetics and Gene Regulation, Department of Otolaryngology-Head and Neck Surgery, Sun Yat-sen Memorial Hospital, Sun Yat-sen University, Guangzhou, China; ^2^ Department of Otolaryngology-Head Neck Surgery, The Sixth Affiliated Hospital of Sun Yat-sen University, Guangzhou, China; ^3^ Department of General Surgery, Nanfang Hospital, Southern Medical University, Guangzhou, China; ^4^ Department of Pathology, The Seventh affiliated Hospital, Sun Yat-sen University, Shenzhen, China; ^5^ State Key Laboratory of Oncology in South China, Collaborative Innovation Center for Cancer Medicine, Sun Yat-sen University Cancer Center, Guangzhou, China

**Keywords:** interferon-stimulated gene 15, tumor-associated microenvironment (TAM), nasopharyngeal carcinoma, tumor microenvironment (TME), prognosis

## Abstract

Interferon-stimulated gene 15 (ISG15) is known to be involved in tumor progression. We previously reported that ISG15 expressed on nasopharyngeal carcinoma (NPC) cells and related to poor prognosis of patients with NPC. We further observed that ISG15 can be secreted by NPC cell and expressed on the macrophages in situ. However, the role of ISG15 in tumor-associated macrophages (TAMs) remains poorly understood. In the present study, we found that ISG15 treatment induces macrophages with M2-like phenotype, and the enhancement of NPC cell migration and tumorigenicity. Mechanically, ISG15-induced M2-like phenotype is dependent on the interaction with its receptor, LFA-1, and engagement of SRC family kinase (SFK) signal, and the subsequent secretion of CCL18. Blocking LFA-1, or SRC signal with small molecular inhibitors, or neutralizing with anti-CCL18 antibody can impede the activation of LFA-1-SFK-CCL18 axis in ISG15-treated macrophages. Clinically, ISG15^+^ CD163^+^ TAMs related to impaired survival of patients and advanced tumor stage of NPC. Furthermore, we found ISG15^+^ CD163^+^ macrophages inhibited antitumor CD8^+^ cells responses in NPC. Together, our findings suggested tumor cell-secreted ISG15, which acted as a tumor microenvironmental factor, induces M2-like phenotype, promoting tumor progression and suppression of cytotoxic T lymphocyte response.

**Graphical Abstract:**
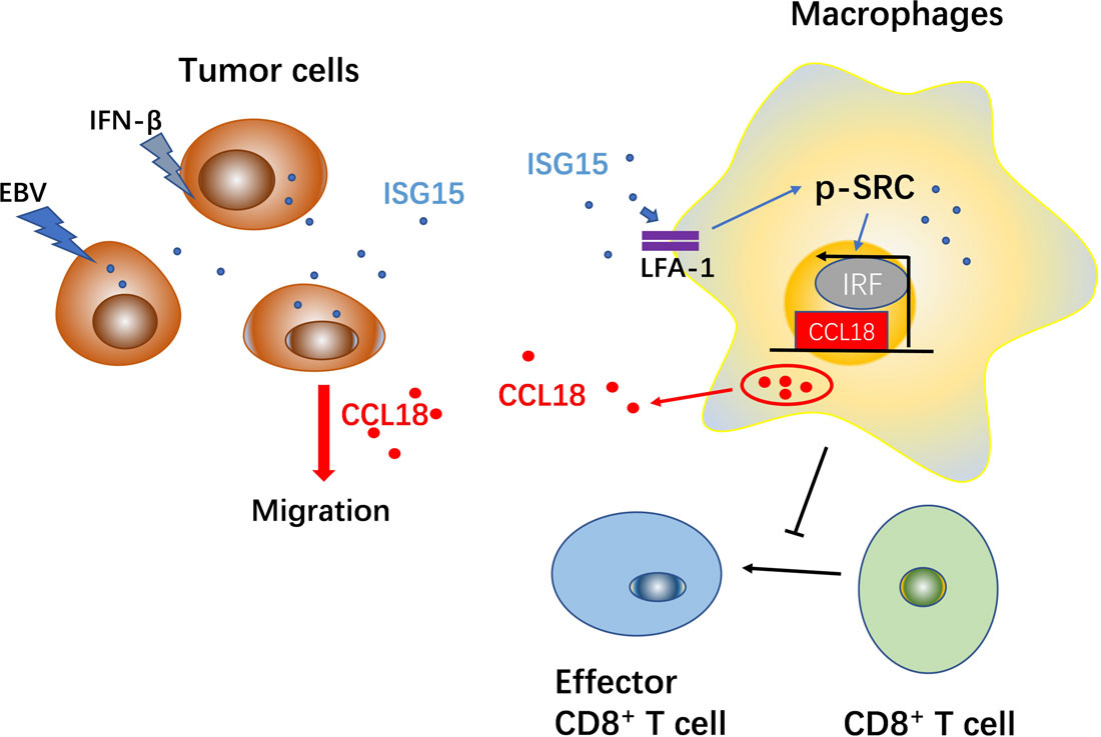


## Introduction

The host innate immune system provides a frontline defense against invading pathogens, and the intensity of this early response can influence the course of disease progression. One of the earliest host responses to viral infection is the production of type I interferons (IFN-α and-β) and the subsequent upregulation of IFN-stimulated genes (ISGs) (1, 2). These ISGs generate an antiviral state and play a central role in regulating the host innate and adaptive immune responses. Among these ISGs, ISG15 is one of the most strongly (3) and rapidly (4) induced genes. ISG15 encodes a small Ubiquitin-like protein of 15 kDa that forms covalent conjugates with cellular proteins mediating robust antiviral responses (5). ISG15 exists in three different forms: unconjugated, conjugated to the target proteins within the cell, and released into the serum (6). When ISG15 is secreted, free ISG15 can function as a cytokine that modulates the immune response. For example, free ISG15 can activate natural killer (NK) cells and enhance lymphokine-activated killer-like activity, stimulate IFN-γ production and induce dendritic cell maturation and neutrophil recruitment (7, 8). However, ISG15 has not been investigated in the context of macrophage differentiation.

Microbial infection causally accounts for more than 20% of malignancies worldwide (9). EBV-encoded RNAs are detected exclusively in tumor cells but not in normal nasopharyngeal epithelium, suggesting that EBV activation is necessary in the pathogenesis of NPC (10).Tumor initiation and progression caused by viral infections reflect properties that are intrinsic to tumor cells, including expression of viral oncoproteins and nontranslated viral RNAs that directly contribute to tumor growth (11), metastasis (12), and treatment resistance (13). In addition, several soluble unregulated EBV factors favor the escape of lymphoma cells from antitumor immune responses while promoting the creation of niches in which tumor cells may find support for their growth and survival (14). Emerging evidence has indicated that persistent viral infections can foster chronic inflammatory microenvironments that extrinsically promote malignant transformation, but their role in NPC is not well established.

Macrophages are major producers of proinflammatory cytokines and are pivotal infiltrates into tumors. Macrophages in tumor tissues have been referred to as tumor-associated macrophages (TAMs), which usually harbor a M2 polarization and confer a protumor phenotype (15). Mounting evidence has indicated that TAMs have complicated dual functions in their interaction with neoplastic cells and are closely associated with poor prognosis in various tumor types (16). TAMs are predominantly detected in both the stroma and tumor cell islets in NPC specimens and are significantly correlated with the survival of NPC patients (17). However, little is known about how macrophages infiltrated into the NPC microenvironment acquire protumor phenotype, and subsequently affect tumor cells and contribute to the progression of NPC. In addition, the interaction between chronic EBV infection and the tumor-promoting inflammatory microenvironment in NPC remains elusive.

We had reported that ISG15 expressed on nasopharyngeal carcinoma (NPC) cell and relates to poor prognosis of patients with NPC (18). Further study indicated ISG15 can be secreted by NPC cells and expressed on the macrophages in NPC tissues, suggesting the regulatory role of ISG15 on tumor-associated macrophages. In this study, we showed that NPC cells released ISG15, which activates macrophages and subsequent CCL18 secretion through LFA-1 and SRC family kinases (SFKs). ISG15-treated macrophages promoted NPC cell migration and were inhibited by a CCL18 neutralizing antibody and LFA-1 or SFK inhibitors. Clinically, ISG15-expressing TAMs related to impaired prognosis in NPC patients. In addition, ISG15-treated macrophages impaired cytotoxic T lymphocyte (CTL) response. These findings suggest that ISG15 might be a microenvironmental (TME) factor linking chronic EBV infection and tumor progression in NPC.

## Materials and Methods

### NPC Specimens

The tissue microarray (TMA) of 90 NPC biopsy specimens between September 2016 and November 2018 were obtained from the Department of Otolaryngology, Head ∧Neck Surgery, Sun Yat-sen Memorial Hospital of Sun Yat-sen University. This cohort of NPC specimens was used for IHC double staining of ISG15 and CD163 to identify the prognostic prediction of ISG15 expressing TAMs in NPC. The informed consent was obtained from each patient prior to biopsy and the study was approved from the Institute Research Ethics Committee. The characteristics of the cohort of NPC patients were included in **Table 1**.

**Table 1:** Univariate and multivariate analysis of different prognostic variables in 90 patients with nasopharyngeal carcinoma.

Variable	All cases	Overall survival				Disease-free survival			
		Univariate analysis		Multivariate analysis		Univariate analysis		Multivariate analysis	
		Mean survival (months)	*p* value	Hazards ratio (95% CI)	*p* value	Mean survival (months)	*p* value	Hazards ratio (95% CI)	*p* value
Sex Female Male Age ≤45 >45	21 69 39 51	36.3 36.9 38.4 38.5	0.678 0.303		– –	36.3 39.1 37.3 38.3	0.572 0.469		– –
T classification			0.206	1.121 (0.772−2.093)	0.346		0.121	1.176 (0.698−1.984)	0.542
T1	15	38.3				38.3			
T2	39	41.8				41.8			
T3	26	36.9				34.8			
T4	10	32.5				32.5			
N classification			0.051	2.159 (1.195−3.901)	0.011		0.013	2.576 (1.388−4.78)	0.003
N0	12	46.8				46.8			
N1	22	39.3				39.3			
N2	45	36.2				35.9			
N3	11	35.5				31.5			
Clinical stage			0.161	0.396 (0.098−1.596)	0.193		0.042	0.584 (0.142−2.405)	0.456
I−III	71	41.3				41.3			
IV	19	35.7				32.6			
Distant metastasis			0.003	1.987 (0.547−7.22)	0.297		0.003	2.148 (0.624−7.394)	0.225
No	83	41.3				40.7			
Yes	7	31.9				29			
CD163^+^ cells			0.058	1.679 (0.524−5.378)	0.383		0.033	2.178 (0.706−6.719)	0.176
Low	45	42.5				39.2			
High ISG15^+^ cells Low	45 44	35.4 42.5	0.032	0.298 (0.028−3.198)	0.318	37.7 42.5	0.02	0.433 (0.046−4.091)	0.465
High ISG15^+^CD163^+^ cells Low High	46 45 45	35.9 42.1 35.4	0.021	2.812 (1.083−7.301)	0.034	34.6 42.1 34.1	0.011	3.244 (1.253−8.398)	0.015

### Cell Culture

The NPC cell lines (C666-1, HK1) were kindly provided by Prof. Qian Zhong at SYSUCC. The cells were cultured in RPMI 1640 (Invitrogen) supplemented with 10% fetal bovine serum (FBS; HyClone) in a humidified 5% CO_2_ incubator at 37°C. All the cells were tested for mycoplasma contamination and authenticated with STR profiling, which was described in **Supplementary Table 1**. Human peripheral blood mononuclear cells (PBMCs) were isolated from the donated anticoagulated peripheral blood by density gradient centrifugation. PBMC-derived monocytes/macrophages were obtained from the CD11b^bright^ cells by using magnetic cell sorting with CD11b microbeads (Miltenyi Biotec, catalog no. 130-049-601). After culturing in RPMI 1640 (Invitrogen) at a concentration of 1×10^6^ cells/ml for 24 h, the adherent cells were macrophages and continued to be cultured in RPMI 1640 supplemented with 10% fetal bovine serum (FBS; HyClone) and 40 ng/ml M-CSF (Novoprotein, catalog no. CB34) in a humidified 5% CO2 incubator at 37°C for 5 days. At this point, the macrophages are mature and used in subsequent experiments. The M1 phenotype was induced by culturing matured macrophages for 2 days in the presence of 1×10^3^ U/ml human IFN-γ (Procell, catalog no. PCK062) prior to the treatment of 25 ng/ml LPS for the last 24 h. The M2 phenotype was generated using 10 ng/ml IL-4 (Novoprotein, catalog no. CD88) for 2 days. CD8 microbeads (Miltenyi Biotec, catalog no. 130-045-201) were used for positive selective of human CD8^+^ T cells from PBMCs. CD8^+^ cells were cultured in the pre-bound plate with 5 ng/ml anti-human CD3 SAFIRE Purified (Biogems, Catalog no. 05121-25-500) for 24 h, subsequently primed in the presence of 5 ng/ml anti-human CD28 SAFIRE Purified (Biogems, Catalog no. 10311-25-500) for 2 days, and then treated with conditional media of ISG15-treated macrophages for 24 h. Induced CD8+ cells were activated with 10 ng/ml BFA for 6 h prior to fluorescence activated cell sorting (FACS) analyses. Recombinant full length ISG15 (Abcam, catalog no. ab78929) was added into the conditional media of macrophages at a dose with range from 0.1 to 0.5 µg/ml. The concentration of LPS in recombinant ISG15 is 0.56 EU/µg and less than requirement of 1 EU/µg *in vitro* cell experiment. Heat-inactivated recombinant ISG15 protein was boiled within 100°C for 15 min and served as a control setting.

### Isolation of Single Cells From Human NPC Samples

Single tissue cells were isolated by using a previously reported method with modification (19). Briefly, human NPC biopsy samples were carefully cleaned and cut into small pieces (<0.5 cm). The tissue fragments were digested with an enzyme mixture of 1 mg/ml collagenase D (Roche Diagnostic), 100 ug/ml DNase I (Sigma-Aldrich), and 0.6 U/ml dispase (Roche Diagnostic) in complete DMEM containing 2% FBS for 60 min. Then, the fragments were filtered, counted and stained for flow cytometric analyses.

### Quantitative Real-Time PCR

Total RNA was extracted using TRIzol (Invitrogen) according to the manufacturer’s instructions. RT-PCR was performed with a Light Cycler 480 system (Roche Diagnostics) using a SYBR Premix ExTaq kit (Takara). The oligonucleotide sequences of quantitative real-time PCR (qRT-PCR) primers are listed below.

IFN-α: forward AACTCCCCTGATGAATGCGG, reverse AGTGTAAAGGTGCACATGACG

IFN-β: forward GCGACACTGTTCGTGTTGTC, reverse GCCTCCCATTCAATTGCCAC

IL-10: forward CGAGATGCCTTCAGCAGAGT, reverse GGCAACCCAGGTAACCCTTA

TGF-β: forward CACGTGGAGCTGTACCAGAA, reverse AGTGAACCCGTTGATGTCCA

iNOS: forward CGCATGACCTTGGTGTTTGG, reverse CATAGACCTTGGGCTTGCCA

### Western Blot Analysis

Protein extracts were resolved by 10% SDS–PAGE, transferred to PVDF membranes (Roche), and probed with antibodies directed against human ISG15 (1:1,000; Abnova, catalog no. A155801), SRC (1:1,000; CST, catalog no. 9935T), and β-actin (1:3,000; Abcam, catalog no. ab69512). Peroxidase-conjugated secondary antibodies (1:3,000; CST) were used.

### Immunoprecipitation

The conditional culture medium (CM) of HK1 and C666-1 NPC cells was filtered with a 0.45 µm syringe filter. An antibody against human ISG15 (Abnova, catalog no. A155801) was added to the filtered CM at a ratio of 1µg:2 ml and shaken for 2 h at 4°C. Protein A/G beads (Pierce protein A/G agarose) were used to precipitate the anti-ISG15 complexes, which were centrifuged, denatured, and analyzed using Western blotting to determine ISG15 expression.

### Double Immunostaining of NPC Specimens

Core tissues that were 2 mm in diameter were obtained from the representative formalin-fixed paraffin-embedded NPC samples were sectioned at 4-µm thickness. Antigen retrieval was performed using a pressure cooker for 30 min in 0.01 mol/L citrate buffer (pH 6.0). The specimens were incubated with antibodies specific for CD163 (1:200; Abcam, catalog no. ab182422) and ISG15 (1:100; Novus, catalog no. NB600-891). Two independent pathologists (T-Z Zhang, L Zhang) who were blind to the clinical status of the patients counted the stained numbers of CD163^+^, ISG15^+^, and ISG15^+^CD163^+^ cells in the intratumoral area under a microscope. For immunofluorescence staining, the formalin-fixed paraffin-embedded specimens of NPC were incubated with antibodies specific for CD163 (1:100; Abcam, catalog no. ab182422) and ISG15 (1:100; Novus, catalog no. NB600-891), or the macrophages slide were incubated with antibodies specific for ISG15 (1:100; Novus, catalog no. NB600-891) and CD11+CD18 (1:100; Abcam, catalog no. ab13219) overnight at 4°C, followed by incubation with Alexa Fluor 555 donkey anti-rabbit IgG and Alexa Fluor 488 donkey anti-mouse IgG (Life Technologies). Cells stained with the indicated antibody were imaged using a confocal laser-scanning microscope (Carl Zeiss) with a core data acquisition system.

### Flow Cytometry Staining

Macrophages with or without rISG15 treatment for 12 h were stained with PE mouse anti-human CD163 (BD Bioscience, catalog no. 556018) and FITC mouse anti-human HLA-DR (BD Bioscience, catalog no. 560944). After gating the macrophages, a two-parameter density blot was used to distinguish M2 and M1 cells by creating a plot on CD163 (PE) and HLA-DR (FITC). For detecting the effectors of CD8^+^ cells, CD8^+^ cells were stained with PE anti-human CD8a (Biolegend, catalog no. 344705) prior to the intracellular staining with PE/Cyanine 7 anti-human IFN-γ (Biolegend, catalog no. 502527), or FITC anti-human/mouse Granzyme B (Biolegend, catalog no. 515403), or APC anti-human Perforin (Biolegend, catalog no. 308111). The intracellular staining permeabilization wash buffer (Biolegend, catalog no. 421002) is used to permeabilize CD8^+^ cells and following fixation with fixation buffer (Biolegend, catalog no. 420801). All data were collected using a cytoFLEX flow cytometer (BD Biosciences, USA) and analyzed using FlowJo software (BD Biosciences, USA).

### *In Vivo* Tumorigenesis and Treatment

To determine the *in vivo* tumorigenicity of ISG15-treated macrophages in NPC, we used a mixture of NPC cells and macrophages to develop a tumor model. A total of 125 μl of a mixture of RPMI 1640 (Invitrogen) and basement membrane matrix (at a ratio of 2:1, Matrigel; Corning, catalog no. 354248) containing 1.5×10^6^ HK1 NPC cells and 0.5×10^6^ human PBMC-derived macrophages was injected subcutaneously into the flanks of female BALB/c mice (5–6 weeks old, Beijing Vital River Laboratory Animal Technology Co., Ltd.). The treatment protocol followed the guidelines for animal experimentation adopted by SYSUCC, and meets the standards required by the UKCCCR guidelines (20). The animal experiments were approved by the Institutional Animal Care and Use Committee at SYSUCC. Macrophages were induced with or without 295 µM rISG15 for 24 h before inoculation. Mice were euthanized using Isoflurane (Batch No. 217150301, RWD Life Science. Co. Ltd, Shenzhen, China) inhalation in their home cages, followed by cervical dislocation to ensure death on two weeks after tumor cell injection. Further information was described in **Supplementary File**.

### Cytokine Array and Cytokine Detection

The supernatants of macrophages treated with or without rISG15 were incubated with detection membranes at 4°C overnight. The procedure was performed according to the manufacturer’s protocol for the RayBio Human Cytokine Antibody Array (Raybiotech, catalog no. AAH-CYT-1000). The protein levels of M1- and M2-polarizing cytokine were detected by using Human TH1/TH2/TH17 Cytokine CBA Kit (BD Bioscience, Catalog no. 560484), which contained a cytokine panel of IL-2, IL-4, IL-6, IL-10, TNF-α, and IFN-γ. The protein level of CCL18 was measured by using Human CCL18/PARC DuoSet ELISA Kit (R&D, Catalog no. DY394-05). The procedures were performed according to the manufacturer’s protocols.

### Statistical Analysis

Student’s t-test was used to compare two independent groups of data. Survival distributions were estimated using the Kaplan–Meier method and the relationship between survival and parameter was analyzed with the log-rank test. Cox proportional hazard regression analyses were performed to identify predictors of prognosis, including factors with a p-value 0.15 in univariate analyses. P<0.05 was considered significant. Statistical analyses were performed using SPSS version 25.0 (SPSS, Chicago, IL, USA) statistical software.

### Data Availability

The data that support the findings of this study are available from the corresponding author upon reasonable request.

## Results

### Nasopharyngeal Carcinoma Cells Released ISG15 Into the Extracellular Environment, and ISG15 Has the Potential to Become a Microenvironment Factor for NPC

**Figure 1:**
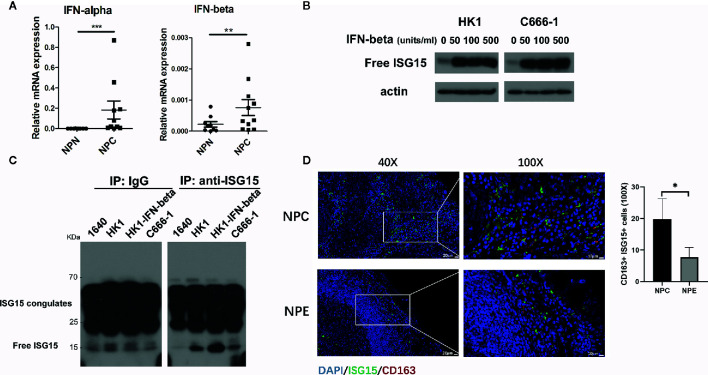
Interferon-stimulated gene 15 (ISG15) is released from nasopharyngeal carcinoma (NPC) cells and functions as a cytokine for macrophages in NPC. **(A)** Upregulated interferon (IFN)-α and β mRNA were detected in NPC biopsy tissues. **(B)** Increased ISG15 protein was determined in HK1 and C666-1 NPC cells treated with IFN-β. **(C)** SDS-PAGE analysis of immunoprecipitated free ISG15 protein from conditional media (CM) of NPC cells. Control as RPMI-1640 CM, and HK1-IFN-β as HK1 cells treated with 50 unit/ml IFN-β for 16 h, and C666-1 as EBV-positive NPC cells. **(D)** Representative confocal images showing double staining of ISG15 and CD163^+^ in NPC and non-cancerous nasopharyngeal epithelium (NPE) sample (left panel). Scale bars, 20 μm in 40X or 10 μm in 100X. The quantification of the number of ISG15^+^ CD163^+^ cells in NPC (n=6) and in NPE (n=3) (right panel). Data are expressed as mean ± SD. Statistical significance was calculated using two-tailed Student’s t test and is indicated by *p < 0.05, **p < 0.01, and ***p < 0.001.

We reported that ISG15 mRNA was upregulated in NPC tissue (18). ISG15 induced the stemness phenotype in NPC cells and was associated with an inferior prognostic of NPC patients (18). As ISG15 is an antiviral protein and induced by type I interferon, we measured the expression of IFN-α and IFN-β mRNA in NPC samples. The results showed that IFN-α and IFN-β mRNA were increased in NPC tissues and suggested that the upregulated ISG15 in NPC tissue may be a response to the increased level of type I IFN (**Figure 1A**). We further detected the ISG15 protein level in NPC cells treated with IFN-β and observed an increased ISG15 in IFN-β-induced NPC cells (**Figure 1B**). ISG15 is a well-known intracellular ubiquitin-like molecule involved in ISGylation. Moreover, free ISG15 can be released into the extracellular space and serves as a cytokine. We detected ISG15 protein in the supernatants of HK1 and EBV^+^ C666-1 NPC cells. SDS-PAGE analysis of immunoprecipitation (IP) in the supernatants suggested that ISG15 can be released by NPC cells (**Figure 1C**). Free ISG15 has been reported to active NK cells (21) and T cells (22), but its effect on macrophages is not known. In nasopharyngeal carcinoma tissues, the number of ISG15^+^ CD163^+^ macrophages were significantly higher than that of non-cancerous nasopharyngeal epithelium (**Figure 1D**), suggesting the infiltration of ISG15^+^ CD163^+^ macrophages relate to the development of NPC. Our results provide evidence for secreted ISG15 from NPC cells has potential to become a microenvironmental factor and have a regulatory effect on macrophages. Next, we study the role of secreted ISG15 on macrophages and acquisition of protumor phenotype.

### ISG15 Induced M2-Like Phenotype and Promoted the Migration and Tumorigenicity of NPC Cells

**Figure 2:**
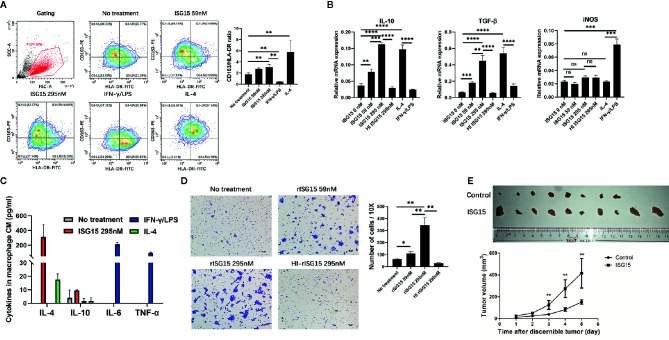
Interferon-stimulated gene 15 (ISG15) induced the M2-like phenotype and promoted NPC cell migration and tumorigenicity. **(A**, **B)** Human peripheral blood mononuclear cell (PBMC)-derived macrophages treated with 59 nM and 295 nM rISG15 for 24 h, and then were detached for fluorescence activated cell sorting (FACS) analysis, or were extracted mRNA for qPCR analysis. **(A)** Representative data of the switched surface markers profile of M2 (CD163)/M1 (HLA-DR) in ISG15-treated macrophages measured by FACS. **(B)** rISG15 induced macrophage mRNA expression of M2 cytokines, such as IL-10 and TGF-β, but not of M1 cytokines, such as iNOS, by qPCR analysis. IFN-γ/LPS and IL-4 served as positive control for M1 and M2 polarization. Heat-inactivated recombinant ISG15 (HI ISG15) protein was boiled within 100°C for 15 min. **(C)** Cytokine panel indicated the increased secretion of M2-polarizing cytokine IL-4 in culture supernatants of ISG15-treated macrophages, but not of M1-polarizing cytokine IL-6 and TNF-α. The culture supernatants were harvested from the macrophages upon rISG15 treatment at indicated dose for 24 h and replaced with serum-free culture for 24 h. **(D)** Representative photography of transwell filters in HK1 NPC cells cultured with the culture medium (CM) of macrophages. Quantification analysis revealed the increased number of transwell cell in the presence of ISG15 treatment. Data are expressed as mean ± SD of three independent experiments. *p < 0.05, **p < 0.01, ***p < 0.001, and ****p< 0.0001. **(E)** Photographic illustration of the grow tumors established by a tumor model within BALB-c mice through subcutaneous inoculation with a mixture of human HK1 NPC cells and PBMC-derived macrophages (at ratio of 3:1) with or without rISG15 pre-treatment. All (10 in 10) mice with ISG15 treatment had tumor grow, but only 8 of 10 mice in the control group had tumor grow. The growth curve showed a significant larger tumor volume in the ISG15 treatment group compared with that of the control. ns, not significant.

Macrophages differentiate into two polarization, M1 and M2. In the tumor microenvironment, tumor-associated macrophages (TAMs) often exhibit the M2 polarization and are associated with malignant transformation of tumor cells and poor prognosis of patients. First, free recombinant ISG15 protein was added into the supernatant of human macrophages derived from peripheral blood mononuclear cells (PBMCs). The surface markers of M2/M1 macrophages, the CD163/ HLA-DR ratio, were measured by flow cytometry. Increased ratio of CD163/ HLA-DR by human macrophages were observed with rISG15 treatment (**Figure 2A**), suggesting ISG15 treatment induces the M2-like phenotype. In addition, ISG15 treatment significantly upregulated the mRNA expression of M2-related cytokines IL-10, TGF-β, but not the M1-related cytokine iNOs in human macrophages (**Figure 2B**). Accordingly, ISG15 treatment increased the secretion of IL-4 (the prototypical M2-polarizing cytokine), without any effector on M1-polarizing cytokines such as IL-6 and TNF-α (**Figure 2C**). Furthermore, the supernatants from the macrophages treated with rISG15 markedly promoted the migration of HK1 NPC cells (**Figure 2D**). Moreover, enhanced tumorigenicity was indicated within BALB/c mice through subcutaneous inoculation of a mixture of NPC cells and ISG15-pretreated macrophages (**Figure 2E**). Taken together, ISG15-activated macrophage enhanced the migration and tumorigenicity of NPC cells.

### ISG15-Treated Macrophages Promoted NPC Cell Migration Through CCL18 Secretion

**Figure 3:**
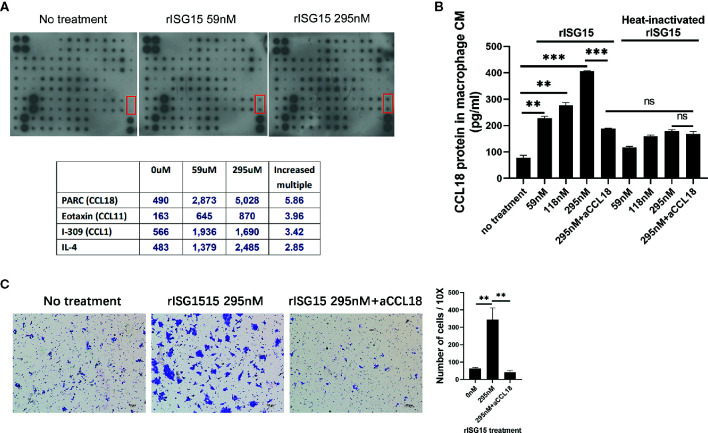
Interferon-stimulated gene 15 (ISG15)-treated macrophages promoted nasopharyngeal carcinoma (NPC) cell migration through CCL18 secretion. **(A)** Cytokine array of the CM of macrophages induced with rISG15 at the indicated doses for 24 h. The lower table summarizing the relative signal intensity of the indicated cytokines is presented. The red marker box indicates the site of CCL18. **(B)** Increased level of CCL18 in the supernatant of ISG15-treated macrophages was confirmed by ELISA. Macrophages were treated with the indicated dose of rISG15 for 24 h. The CCL18-inducing effect of ISG15 was blocked by pretreatment with a CCL18 neutralizing antibody, and was not observed in heat-inactivated rISG15. **(C)** The increased number of infiltrated NPC cells, as measured by transwell assay, in the presence of CM from ISG15-treated macrophages. The enhancement was abolished by anti-CCL18 antibody. Data are expressed as mean ± SD of three independent experiments. **p < 0.01 and ***p < 0.001.

To determine how the ISG15-treated macrophages promote NPC cell migration, the cytokine profile in the supernatants of macrophages treated with ISG15 was examined by using a cytokine array (**Supplementary Tables 2** and **3**). The results indicated that CCL18 is the cytokine with the highest increase in the supernatants of ISG15-treated macrophages compared with that of the control (**Figure 3A**). We confirmed the increased secretion of CCL18 in ISG15-treated macrophages by ELISA (**Figure 3B**). These results suggested that secreted ISG15 is a CCL18-inducing molecule in macrophages. CCL18 has been shown to be exclusively secreted by myeloid-derived cells and is one of the most abundantly produced cytokines of TAMs (23, 24). TAM-derived CCL18 has been reported to promote cancer cell invasion by inducing the epithelial–mesenchymal transition (EMT) (23, 25, 26). Recently, CCL18 was confirmed to induce EMT in NPC (27). In our study, the enhanced migration of NPC cells induced by the supernatants of ISG15-treated macrophages was abolished in the presence of polyclonal anti-CCL18 antibodies (**Figure 3C**). Taken together, ISG15-treated macrophages promoted NPC cell migration through CCL18 secretion.

### ISG15-Induced CCL18 Secretion by Macrophages Was Dependent on the ISG15 Receptor, Leukocyte Function-Associated Antigen-1 (LFA-1), and SRC Family Kinase Signaling

**Figure 4:**
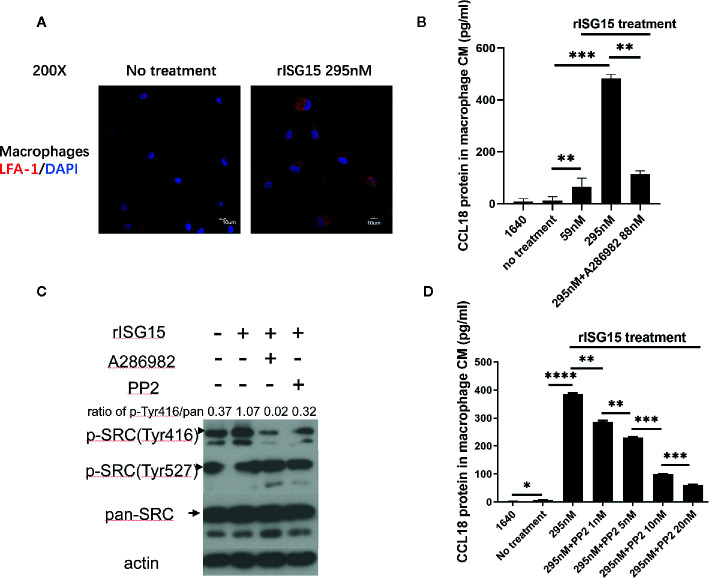
Interferon-stimulated gene 15 (ISG15)-induced CCL18 secretion by macrophages was dependent on the ISG15 receptor, leukocyte function-associated antigen-1 (LFA-1), and SRC family kinase (SFK) signaling. **(A)** Immunofluorescence demonstrated that the ISG15 receptor CD11a/CD18, also called LFA-1, was expressed on the membranes of human macrophages. Macrophages were treated with 295nM rISG15 for 24 h. Scale bars, 10 μm. **(B)** ELISA results showed small molecular inhibitors of LFA-1, A286982, inhibited CCL18 secretion by macrophages. **(C)** Small molecular inhibitors of LFA-1 and SRC, A286982 and PP2, hindered the ISG15-induced activation of SFK signaling. **(D)** PP2 reduced the CCL18 secretion by ISG15-treated macrophages in a dose-dependent manner, as determined by ELISA. *p < 0.05, **p < 0.01, ***p < 0.001, and ****p < 0.0001.

CD11a (αL integrin) forms a heterodimeric complex with CD18 (β2 integrin) to form the leukocyte function-associated antigen-1 (LFA-1) integrin receptor. In NK cells, LFA-1 has been reported to be the receptor for ISG15 and that the αl domain of CD11a contains the binding site for ISG15 (21). We determined whether LFA-1 is the same receptor for ISG15 in macrophages. LFA-1 expression was detected on the membranes of human macrophages treated with ISG15 (**Figure 4A**). A small molecular inhibitor of LFA-1, A286982, inhibited CCL18 secretion by macrophages (**Figure 4B**). These results verified that the ISG15-induced CCL18 secretion by macrophages was dependent on LFA-1, the receptor for ISG15. As ISG15 treatment increased the IL-10 secretion of NK cells by SRC signaling (21), we examined whether ISG15-treated macrophages consistently activate SRC signaling. We observed small molecular inhibitors of LFA-1 and SRC, A286982 and PP2, hindered the activation of ISG15-induced SRC signaling (**Figure 4C**). The activation of SRC signaling by phosphorylation of Tyr416 in a time-dependent manner in ISG15-treated macrophages (**Supplementary Figure 1A**). Moreover, PP2 reduced the CCL18 secretion by ISG15-treated macrophages in a dose-dependent manner (**Figure 4D**). Taken together, the ISG15-induced CCL18 secretion by macrophages was dependent on the ISG15 receptor LFA-1 and SRC signaling. In addition, the enhanced migration of NPC cells induced by the supernatants of ISG15-treated macrophages was impeded in the present of LFA-1 inhibitor (**Supplementary Figure 1B**). These observations suggested ISG15-treated macrophages promote NPC cell migration depend on the LFA-1-SRC-CCL18 axis.

### ISG15-Treated Macrophages Exhibited Increased Intracellular ISG15 Expression and ISG15^+^ CD163^+^ TAMs Predicted Poor Survival of NPC Patients

**Figure 5:**
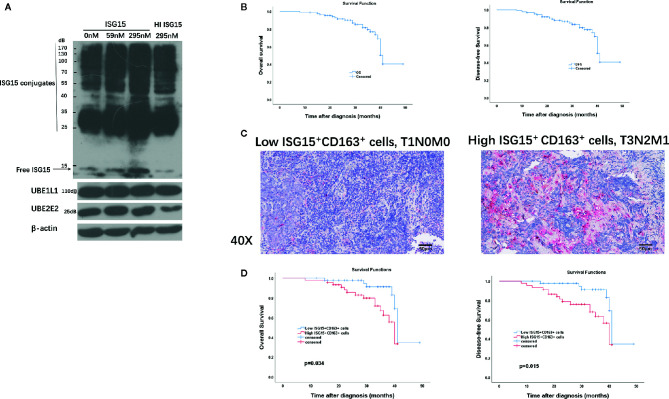
Clinical implication of tumor-associated macrophages (TAMs) and ISG15-expressing TAMs in NPC. **(A)** Intracellular free and conjugated ISG15, and ISGytion E1 enzyme (UBE1L) and E2 enzymes (UBE2E2) were assessed by Western blotting on total cell lysates of rISG15 treated macrophages. **(B)** Kaplan-Meier survival curve of overall survival (OS) and disease-free survival (DFS) in the TMA contained 90 NPC biopsy specimens. **(C)** Representative immunohistochemistry data with double staining of CD163 (in wine red) and ISG15 (in brown) in early (T1N0M0) and advanced (T3N2M1) Tumor Node Metastasis (TNM) stage in NPC samples. Scale bars, 50 μm. **(D)** Kaplan-Meier survival curve of OS and DFS in NPC patients with distinct ISG15^+^ CD163^+^ TAMs count.

We were intrigued whether extracellular ISG15 may upregulate intracellular ISG15 expression in macrophages. The results revealed that intracellular expression of ISG15 increased with the treatment of rISG15 (**Figure 5A**). Furthermore, the increased intracellular ISG15 was mainly in free ISG15 manner, rather than conjugated ISG15, and ISG15 treatment did not activate ISGytion by upregulating E1 activating enzyme (UBE1L1) and E2 enzyme (UBE2L2) in macrophages (**Figure 5A**). Thus, secreted ISG15 treatment upregulated intracellular free ISG15 expression in macrophages.

Immunohistochemistry (IHC) was performed in an independent formalin fixed, paraffin-embedded-based (FFPE-based) TMA consisting of 90 NPC biopsy samples. The included NPC patients were diagnosed at Sun Yat-sen Memorial Hospital of Sun Yat-sen University between September 2016 to November 2018 with complete follow-up until February 2020. Sixty-nine (76.7%) patients were alive at last follow-up. The median OS for all patients from diagnosis was 31 months (range, 8–49 months). The median DFS for all patients was 31 months (range, 8–49 months). The OS and DFS curves of all NPC patients were presented in **Figure 5B**. The 2-, 3-year OS rates are 91.6% and 76.9%, respectively; the 2-, 3- year DFS rates are 88.1% and 74%, respectively.

CD163 is a specific marker for TAMs (16, 28). We performed double immunostaining of ISG15 and CD163 in the cohort of NPC biopsy samples for analyzing the correlation between numbers of ISG15^+^ TAM and NPC patients’ prognosis. Out of 90 NPC cases, 87 specimens (96.6%) expressed CD163^+^ cells, 57 specimens (63.3%) stained ISG15 mainly in tumor cells and infiltrating immune cells, and 49 specimens (54.4%) expressed ISG15^+^ CD163^+^ cells, which suspected to be macrophages based on their morphology as well (**Figure 5C**). The median number of CD163^+^, ISG15^+^, ISG15^+^ CD163^+^ cells per 1 mm^2^ were 132.5 (range 0-506), 6 (range 0–95), 2.5 (range 0–78), respectively.

The median number was used as cutoff to divide specimens into high and low expression groups. As shown in **Table 1**, patients with high ISG15^+^ cells or high ISG15^+^ CD163^+^ TAMs showed a significant worse overall survival (OS) in univariant analysis (*p*=0.032; *p*=0.021, respectively), and patients with high CD163^+^ TAMs tended to have an impaired OS (*p*=0.058). In addition, increased CD163^+^, ISG15^+^, and ISG15^+^ CD163^+^ cells were associated with worse disease-free survival (DFS) in univariant analysis (*p*=0.033; *p*=0.02; *p*=0.011, respectively). Further analysis with a Cox proportional hazards model was performed to determine whether ISG15^+^ CD163^+^ TAMs could serve as an independent prognostic predictor. A series of predictive factors, including T classification, N classification, distant metastasis, expression level of CD163^+^, ISG15^+^ and ISG15^+^ CD163^+^ cells, were included in the multivariate Cox regression analysis. The multivariate analysis model showed that ISG15^+^ CD163^+^ cells were the predominant independent predictors of OS (Hazards ratio, 4.5; *p*=0.034) and DFS (Hazards ratio, 5.9; *p*=0.015) (**Figure 5D**). Other survival predictors were summarized in **Table 1**.

The number of ISG15^+^ CD163^+^ TAMs was significantly associated with advanced clinical stage, IV stage of NPC as compared with I-III stage (*p*=0.016). However, there were no evident correlations with tumoral (T) stage, lymph nodal (N) stage and distant metastasis (M) stage (*p*>0.05) (**Supplementary Figure 2**).

### Higher Expression of ISG15^+^ CD163^+^ Macrophages in NPC Inhibited Antitumor CD8^+^ Cells Responses

**Figure 6:**
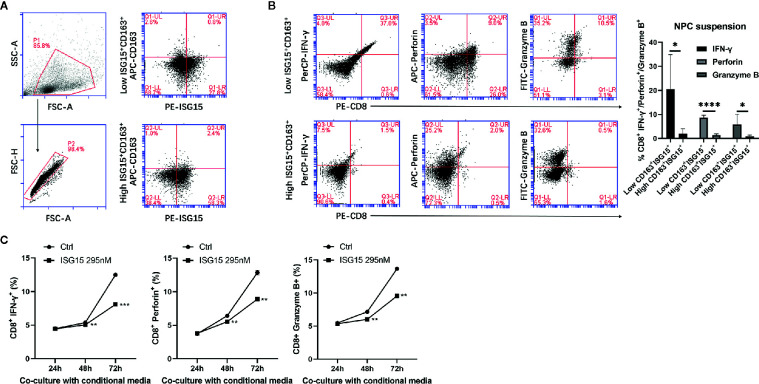
ISG15^+^ CD163^+^ TAMs suppressed cytotoxic leukocyte (CTL) response in nasopharyngeal carcinoma (NPC). **(A)** Single cell suspensions from NPC tissues (n=9) were stained with antibodies for ISG15-PE and CD163-APC. Total frequency of ISG15^+^ CD163^+^ cells were determined by FACS analysis and were indicated as percentage in upper right quadrant. Left penal showed single cell suspensions of NPC tissue were gated. Right panel showed representative FACS photograph of low or high CD163^+^ ISG15^+^ cells. **(B)** Representative FACS analysis and quantification of the percentage of IFN-γ^+^, perforin^+^, and Granzyme B^+^ cells within CD8^+^ T cells between high and low CD163^+^ ISG15^+^ cells groups in NPC tissue suspensions. **(C)** Flow cytometric quantification of the percentage of IFN-γ^+^, perforin^+^, and Granzyme B^+^ cells within CD8^+^ T cells *in vitro* cocultured with or without the conditional media from ISG15-treated macrophages over the indicated time. *p < 0.05, **p < 0.01, ***p < 0.001, and ****p < 0.0001.

Accumulation of macrophages in many types of tumors are thought to regulate every step of tumor development, including antitumor T cell responses. Since cytotoxic CD8^+^ T cell has cytotoxic activity against tumor cells, we determined whether ISG15^+^ TAMs may impair the intrinsic functionality of CTLs. The total frequency of ISG15^+^ CD163^+^ cells in suspension of NPC tissues varied from 1% to 9.7% among all live cells in the tumor microenvironment. We used the median frequency of ISG15^+^ CD163^+^ cells as cut-off value to divide NPC tissues into high or low expression of ISG15^+^ TAMs groups (**Figure 6A**). We found that the production of IFN-γ, perforin, and Granzyme B by CD8^+^ T cell is significantly lower in high ISG15^+^ TAMs group as compared with low ISG15^+^ TAMs group (**Figure 6B**). This result suggests ISG15^+^ TAMs infiltrated in the microenvironment of NPC could inhibit the antitumor responses of cytotoxic CD8^+^ cells. Next, we determined the effect of ISG15-treated macrophages on the production of IFN-γ, perforin and Granzyme B by CD8^+^ T cells derived from PBMCs after stimulating with plate-bound anti-CD3 and anti-CD28 antibodies for 24 h. We found that the production of IFN-γ, perforin and Granzyme B by CD8^+^ T cells was markedly reduced in the settings of conditional culture medium of ISG15-treated macrophages as compared with non-ISG15-treated macrophages, indicating impaired effector differentiation (**Figure 6C** and **Supplementary Figure 3**). Together, our phenotypic analysis demonstrated that ISG15^+^ TAMs in NPC inhibited the antitumor CTL responses.

## Discussion

ISG15 is released by fibroblasts, monocytes, lymphocytes and neutrophils, and in some of these cell types, secretion may be induced in an IFN-α/β-dependent or IFN-α/β-independent manner (22). Human ISG15-induced PBMCs secrete substantial amounts of IFN-γ, IL-2 and IL-12 (7) and are considered to have cytokine-inducing activity. Moreover, human ISG15 enhances the proliferation and lytic capability of NK cells (7). These findings suggest that ISG15 has extracellular immunomodulating activity. Recently, individuals with ISG15 deficiency have been identified (22, 29). Contrary to expectations, no particular susceptibility to viral infections was observed in these ISG15-deficient patients. The lack of secreted free ISG15 resulted in lower levels of lymphocyte IFN-γ production (22), and the impaired IFN-γ production by blood leukocytes from ISG15-deficient patients leads to susceptibility to environmental mycobacteria (29). These findings suggested that ISG15 is a secreted molecule with a functional role in immunity.

ISG15 can be released as an extracellular molecule in response to type I IFN (7). In this study, NPC cells released ISG15 into the extracellular environment. The mechanism of free ISG15 release or secretion has not been fully elucidated. We observed increased free ISG15 protein in IFN-β-induced NPC cells, possibly suggesting the NPC cells that secreted ISG15 may respond to the increased free intracellular ISG15 under the induction of type I IFN. Furthermore, Sainz and colleagues observed macrophages increase the expression and secretion of ISG15 when cocultured with cancer stem cells (30). This observation indicated that beyond the tumor cells, infiltrated macrophages within cancer microenvironment may release ISG15.

TAMs participate in the initiation and progression of tumors. TAMs secrete ISG15, which contributes to the formation of cancer stem cells of pancreatic ductal adenocarcinoma (30). However, the function of ISG15 in macrophages is not well understood. In this study, secreted ISG15 upregulated intracellular ISG15 expression in macrophages. This phenomenon indicates ISG15-labeled macrophages can be served as a biomarker for further study the clinical implication that ISG15 acts on macrophages. We observed that ISG15-expressing TAMs related to poor prognosis and more advanced stage in NPC patients. *In vitro* studies revealed that ISG15 switches the HLA-DR/CD163 ratio and increases the expression of TAM-like cytokines. These results suggested the functional role of human ISG15 on macrophages. Furthermore, ISG15-treated macrophages promoted the migration and tumorigenicity of NPC cells, either *in vitro* or *in vivo*. These findings confirmed the malignant phenotype of ISG15-treated macrophages.

It has been previously delineated that ISG15 is an alarmin that induces tissue alert by extracellular matrix remodeling, myeloid cell infiltration, and inflammation in mice. ISG15 produced at the vaccination site promoted the vaccine-specific CTL response by enhancing expansion, short-lived effector and effector/memory differentiation of CD8^+^ T cells (31). Another *in vivo* study indicates that ISG15 encoded by a DNA vaccine can promote the CTL response in mice (32). In our observation, free ISG15 acts on macrophage subsequently results in a diverse effect on CTL response. ISG15 expressing TAMs impaired antitumor CTL response according to effector production by the digested live cells from tumor samples and *in vitro* activated CD8^+^ cells treated with ISG15-treated macrophages, suggesting ISG15-treated macrophages may contribute to malignant transformation through suppressive regulation on CTL response to tumor.

Cytokines are canonical signals that directly link immune cells and tumor cells. In this study, a cytokine array demonstrated that CCL18 was the cardinal secreted molecule in the medium of ISG15-treated macrophages. CCL18 from TAMs promoted tumor cell EMT and functioned as a crucial microenvironmental factor involved tumor progression (23, 25). Our work reinforced the protumor role of CCL18 on NPC cell migration. This result was consistent with the findings established by Huang et al (27). CCL18 closely correlated with serum EBV infection titers and tumor progression in two cohorts of NPC patients. EBV^+^ NPC cell lines exhibited superior capacity to attract monocytes and skew them to differentiate to a M2 macrophage (27). These findings demonstrated a link between tumor cell migration and EBV-induced chronic infection. Given the antiviral nature of ISG15, it is presumed to be a predominant microenvironmental factor that links EBV-induced chronic infection and TAMs. Moreover, CCL18 from ISG15-treated TAMs may establish a loop between viral infection, TAMs and tumor progression.

LFA-1 has been recently determined as the membrane receptor of ISG15 in NK cells (21). Our work demonstrated the expression of LFA-1 on macrophages, and the inhibition of LFA-1 abolished CCL18 secretion from ISG15-treated macrophages. These results identified that ISG15 functions on macrophages similarly *via* the surface receptor LFA-1. Furthermore, ISG15 engagement of LFA-1 led to the activation of SRC family kinases (SFKs) (21). In our study, ISG15 induced the activation of SRC signaling by phosphorylation of Tyr416 in a time-dependent manner. In addition, the SFK-blocking agent inhibited CCL18 secretion by ISG15-treated macrophages. These findings revealed that LFA-1-SFK signaling is the pathway by which ISG15 functions in TAMs and induces subsequent CCL18 secretion.

In summary, chronic infection with EBV and increased expression of IFN-α and IFN-β may contribute to the upregulation of ISG15 in NPC cells. NPC cells can release ISG15 in an autocrine manner, which promotes the M2-like phenotype. ISG15-treated macrophages secrete CCL18, which promotes the migration of NPC cells. Tumor cells secrete ISG15, and ISG15-activated TAMs promote cancer cell migration which develops a feed-forward loop between NPC cells and TAMs. These findings show a potential link between chronic viral infection and tumor progression.

## Data Availability Statement

The authors acknowledge that the data presented in this study must be deposited and made publicly available in an acceptable repository, prior to publication. Frontiers cannot accept a manuscript that does not adhere to our open data policies.

## Ethics Statement

The studies involving human participants were reviewed and approved by the Ethics Committee of Sun Yat-sen Memorial Hospital of Sun Yat-sen University. The patients/participants provided their written informed consent to participate in this study. The animal study was reviewed and approved by Institutional Animal Care and Use Committee of Sun Yat-sen University Cancer Center (SYSUCC).

## Author Contributions

Conception and design: R-HC, QZ, X-MH. Development of methodology: R-HC, QZ. Acquisition of data (provided animals, acquired and managed patients, provided facilities, etc.): R-HC, Z-WX, X-QY, PH, F-YL, J-YW, S-TY, TZZ. Analysis and interpretation of data (e.g., statistical analysis, biostatistics, computational analysis): R-HC, Z-WX, S-TY, S-QC. Writing, review, and/or revision of the manuscript: R-HC, PH, QZ, X-MH. Study supervision: QZ, X-MH. All authors contributed to the article and approved the submitted version.

## Funding

This work was supported by the grants from the National Natural Science Foundation of China (81672681, 81872193, to X-MH; 81702697, to PH; 81702719, to S-QC), the Natural Science Foundation of Guangdong Province (2017A030313528, to R-HC), and the Key Laboratory of Malignant Tumor Gene Regulation and Target Therapy of Guangdong Province (2020B1212060018). We thank Long Zhang, a pathologist in our institute for his assistance in immunochemistry scoring.

## Conflict of Interest

The authors declare that the research was conducted in the absence of any commercial or financial relationships that could be construed as a potential conflict of interest.

## Abbreviations

CCL18, C-C motif chemokine ligand 18; CTL, cytotoxic leukocyte; CM, culture medium; DFS, disease-free survival; HLA-DR, Human Leukocyte Antigen- DR isotype; IFN, interferon; IHC, immunohistochemistry; IL-10, Interleukin 10; ISG, Interferon-stimulated gene; LFA-1, leukocyte function-associated antigen-1; NK, natural killer; NPC, nasopharyngeal carcinoma; OS, overall survival; PBMC, peripheral blood mononuclear cell; SFKs, SRC family kinases; TAM, tumor-associated macrophage; TGF-β, transforming growth factor beta; TMA, tissue microarray; TNF-α, tumor necrosis factor--α.

## References

[1] Sledz CA Holko M de Veer MJ Silverman RH Williams BR Activation of the interferon system by short-interfering RNAs. Nat Cell Biol (2003) 5(9):834–9. 10.1038/ncb1038 12942087

[2] Sadler AJ Williams BR Interferon-inducible antiviral effectors. Nat Rev Immunol (2008) 8(7):559–68. 10.1038/nri2314 PMC2522268 18575461

[3] Der SD Zhou A Williams BR Silverman RH Identification of genes differentially regulated by interferon alpha, beta, or gamma using oligonucleotide arrays. Proc Natl Acad Sci USA (1998) 95(26):15623–8. 10.1073/pnas.95.26.15623 PMC28094 9861020

[4] Loeb KR Haas AL The interferon-inducible 15-kDa ubiquitin homolog conjugates to intracellular proteins. J Biol Chem (1992) 267(11):7806–13. 1373138

[5] Au WC Moore PA Lowther W Juang YT Pitha PM Identification of a member of the interferon regulatory factor family that binds to the interferon-stimulated response element and activates expression of interferon-induced genes. Proc Natl Acad Sci USA (1995) 92(25):11657–61. 10.1073/pnas.92.25.11657 PMC40461 8524823

[6] Zhao C Denison C Huibregtse JM Gygi S Krug RM Human ISG15 conjugation targets both IFN-induced and constitutively expressed proteins functioning in diverse cellular pathways. Proc Natl Acad Sci USA (2005) 102: (29):10200–5. 10.1073/pnas.0504754102 PMC1177427 16009940

[7] D’Cunha J Knight E Jr. Haas AL Truitt RL Borden EC Immunoregulatory properties of ISG15, an interferon-induced cytokine. Proc Natl Acad Sci USA (1996) 93(1):211–5. 10.1073/pnas.93.1.211 PMC40208 8552607

[8] Recht M Borden EC Knight E Jr. A human 15-kDa IFN-induced protein induces the secretion of IFN-gamma. J Immunol (Baltimore Md 1950) (1991) 147(8):2617–23. 1717569

[9] Hussain SP Harris CC Inflammation and cancer: an ancient link with novel potentials. Int J Cancer (2007) 121(11):2373–80. 10.1002/ijc.23173 17893866

[10] Chua MLK Wee JTS Hui EP Chan ATC Nasopharyngeal carcinoma. Lancet (London England) (2016) 387(10022):1012–24. 10.1016/S0140-6736(15)00055-0 26321262

[11] Qiu J Smith P Leahy L Thorley-Lawson DA The Epstein-Barr virus encoded BART miRNAs potentiate tumor growth in vivo. PloS Pathogens (2015) 11(1):e1004561. 10.1371/journal.ppat.1004561 25590614 PMC4295875

[12] Cai L Ye Y Jiang Q Chen Y Lyu X Li J Epstein-Barr virus-encoded microRNA BART1 induces tumour metastasis by regulating PTEN-dependent pathways in nasopharyngeal carcinoma. Nat Commun (2015) 6:7353. 10.1038/ncomms8353 26135619 PMC4507016

[13] Zheng X Wang J Wei L Peng Q Gao Y Fu Y Epstein-Barr Virus MicroRNA miR-BART5-3p Inhibits p53 Expression. J Virol (2018) 92(23):e01022-18. 10.1128/JVI.01022-18 30209170 PMC6232473

[14] Dolcetti R Cross-talk between Epstein-Barr virus and microenvironment in the pathogenesis of lymphomas. Semin Cancer Biol (2015) 34:58–69. 10.1016/j.semcancer.2015.04.006 25953434

[15] Ostuni R Kratochvill F Murray PJ Natoli G Macrophages and cancer: from mechanisms to therapeutic implications. Trends Immunol (2015) 36(4):229–39. 10.1016/j.it.2015.02.004 25770924

[16] Ruffell B Affara NI Coussens LM Differential macrophage programming in the tumor microenvironment. Trends Immunol (2012) 33(3):119–26. 10.1016/j.it.2011.12.001 PMC3294003 22277903

[17] Liao Q Zeng Z Guo X Li X Wei F Zhang W LPLUNC1 suppresses IL-6-induced nasopharyngeal carcinoma cell proliferation via inhibiting the Stat3 activation. Oncogene (2014) 33(16):2098–109. 10.1038/onc.2013.161 23708661

[18] Chen RH Du Y Han P Wang HB Liang FY Feng GK ISG15 predicts poor prognosis and promotes cancer stem cell phenotype in nasopharyngeal carcinoma. Oncotarget (2016) 7(13):16910–22. 10.18632/oncotarget.7626 PMC4941359 26919245

[19] Sato K Honda SI Shibuya A Shibuya K Cutting Edge: Identification of Marginal Reticular Cells as Phagocytes of Apoptotic B Cells in Germinal Centers. J Immunol (Baltimore Md 1950) (2018) 200(11):3691–6. 10.4049/jimmunol.1701293 29686051

[20] Workman P Aboagye EO Balkwill F Balmain A Bruder G Chaplin DJ Guidelines for the welfare and use of animals in cancer research. Br J Cancer (2010) 102(11):1555–77. 10.1038/sj.bjc.6605642 PMC2883160 20502460

[21] Swaim CD Scott AF Canadeo LA Huibregtse JM Extracellular ISG15 Signals Cytokine Secretion through the LFA-1 Integrin Receptor. Mol Cell (2017) 68(3):581–90.e5. 10.1016/j.molcel.2017.10.003 29100055 PMC5690536

[22] Bogunovic D Byun M Durfee LA Abhyankar A Sanal O Mansouri D Mycobacterial disease and impaired IFN-γ immunity in humans with inherited ISG15 deficiency. Science (2012) 337(6102):1684–8. 10.1126/science.1224026 PMC3507439 22859821

[23] Chen J Yao Y Gong C Yu F Su S Chen J CCL18 from tumor-associated macrophages promotes breast cancer metastasis via PITPNM3. Cancer Cell (2011) 19(4):541–55. 10.1016/j.ccr.2011.02.006 PMC3107500 21481794

[24] Schutyser E Struyf S Proost P Opdenakker G Laureys G Verhasselt B Identification of biologically active chemokine isoforms from ascitic fluid and elevated levels of CCL18/pulmonary and activation-regulated chemokine in ovarian carcinoma. J Biol Chem (2002) 277(27):24584–93. 10.1074/jbc.M112275200 11978786

[25] Su S Liu Q Chen J Chen J Chen F He C A positive feedback loop between mesenchymal-like cancer cells and macrophages is essential to breast cancer metastasis. Cancer Cell (2014) 25(5):605–20. 10.1016/j.ccr.2014.03.021 24823638

[26] Lane D Matte I Laplante C Garde-Granger P Carignan A Bessette P CCL18 from ascites promotes ovarian cancer cell migration through proline-rich tyrosine kinase 2 signaling. Mol Cancer (2016) 15(1):58. 10.1186/s12943-016-0542-2 27613122 PMC5017134

[27] Huang D Song SJ Wu ZZ Wu W Cui XY Chen JN Epstein-Barr Virus-Induced VEGF and GM-CSF Drive Nasopharyngeal Carcinoma Metastasis via Recruitment and Activation of Macrophages. Cancer Res (2017) 77(13):3591–604. 10.1158/0008-5472.CAN-16-2706 28484077

[28] Zhou D Huang C Lin Z Zhan S Kong L Fang C Macrophage polarization and function with emphasis on the evolving roles of coordinated regulation of cellular signaling pathways. Cell Signal (2014) 26(2):192–7. 10.1016/j.cellsig.2013.11.004 24219909

[29] Zhang X Bogunovic D Payelle-Brogard B Francois-Newton V Speer SD Yuan C Human intracellular ISG15 prevents interferon-alpha/beta over-amplification and auto-inflammation. Nature (2015) 517(7532):89–93. 10.1038/nature13801 25307056 PMC4303590

[30] Sainz B Jr. Martin B Tatari M Heeschen C Guerra S ISG15 is a critical microenvironmental factor for pancreatic cancer stem cells. Cancer Res (2014) 74(24):7309–20. 10.1158/0008-5472.CAN-14-1354 25368022

[31] Iglesias-Guimarais V Ahrends T de Vries E Knobeloch KP Volkov A Borst J IFN-Stimulated Gene 15 Is an Alarmin that Boosts the CTL Response via an Innate, NK Cell-Dependent Route. J Immunol (Baltimore Md 1950) (2020) 204(8):2110–21. 10.4049/jimmunol.1901410 PMC7128311 32169846

[32] Villarreal DO Wise MC Siefert RJ Yan J Wood LM Weiner DB Ubiquitin-like Molecule ISG15 Acts as an Immune Adjuvant to Enhance Antigen-specific CD8 T-cell Tumor Immunity. Mol Ther (2015) 23(10):1653–62. 10.1038/mt.2015.120 PMC4817913 26122932

