# Supplementary material for: Tumor Cell-Secreted ISG15 Promotes Tumor Cell Migration and Immune Suppression by Inducing the Macrophage M2-Like Phenotype
Source: Front Immunol. 2020 Dec 23;11:594775. doi: 10.3389/fimmu.2020.594775 (PMC7785797; doi:10.3389/fimmu.2020.594775)
Supplement: Supplementary file 1 [file DataSheet_1.doc]

**Supplementary File**

**Secreted ISG15 acting as a microenvironmental factor promoted tumor progression by inducing tumor-associated microphages phenotype**

**Running title:** **Secreted ISG15 induces tumor-associated macrophages**

Ren-Hui Chen^1*^, Zhi-Wen Xiao^1,3*^, Ping Han^1^, Fa-Ya Liang^1^, Jing-Yi Wang^1^, Shi-Tong Yu^4^, Ting-Zhen Zhang^5^, Qian Zhong^2#^, Xiao-Ming Huang^1#^

^1^ Guangdong Provincial Key Laboratory of Malignant Tumor Epigenetics and Gene Regulation, Department of Otolaryngology-Head and Neck Surgery, Sun Yat-sen Memorial Hospital, Sun Yat-sen University, Guangzhou, China.

^2^ State Key Laboratory of Oncology in South China, Collaborative Innovation Center for Cancer Medicine, Sun Yat-sen University Cancer Center, Guangzhou, China.

^3^ Department of Otorhinolaryngology- Head and Neck Surgery, The Sixth Affiliated Hospital of Sun Yat-sen University, Guangzhou, China.

^4^ Department of General Surgery, Nanfang Hospital, Southern Medical University, Guangzhou, China.

^5^ Department of Pathology, the Seventh affiliated Hospital, Sun Yat-sen University, Shenzhen, China.

*R.-H. Chen and Z.-W. Xiao contributed equally to this article.

**Supplementary Material and Methods**

**In vivo tumorigenesis and treatment**

Female nude mice (BALB/c) at 5-6 weeks of age were purchased from Beijing Vital River Laboratory Animal Technology Co., Ltd. (Beijing, China) and were placed in SPF rooms for feeding and observation on the Seventh floor of the Experimental Animal Laboratory of Sun Yat-sen University Cancer Center (SYSUCC). Mice have free access to water and food, housed in pathogen-free cages containing wood shavings and bedding in a 12-h light/dark cycle, with controlled room temperature. The treatment protocol followed the guidelines for animal experimentation adopted by SYSUCC, and meets the standards required by the UKCCCR guidelines^18^. The mice were randomly divided into two groups, control group and rISG15 treatment group. Each group contains ten mice. A total of 125 μL of a mixture of RPMI 1640 (Invitrogen) and basement membrane matrix (at a ratio of 2:1, Matrigel; Corning, catalog no. 354248) containing 1.5×10^6^ HK1 NPC cells and 0.5×10^6^ human PBMC-derived macrophages was injected subcutaneously into the flanks of mice under Isoflurane inhalation (RWD Life Science. Co. Ltd, Shenzhen, China, Batch No. 217150301) on 1^st^ August, 2019. The concentration of isoflurane was MAC 1.6%. The depth of anesthesia was under control to make sure the well condition of animal during treatment. An oxygen inhalation device was prepared in case of rescue. Macrophages were induced with or without 295 µM rISG15 for 12 hours before inoculation. The mice were observed every 2 days and weighed. The volume of the tumors was measured every 2 days after the growth of the tumors began. The formula for calculating the volume of tumors was as follows: V = (length × width^2^)/2. Mice were euthanized using Isoflurane inhalation in their home cages, followed by cervical dislocation to ensure death on two weeks after tumor cell injection. The tumors were photographed and stored at -80 ℃.

**Supplemental table 1. The STR profiling of Hela cell line and HK1, C666-1 cell lines**

|  | AMEL | D5S818 | D13S317 | D7S820 | D16S539 | vWA | THO1 | TPOX | CSF1PO |
| --- | --- | --- | --- | --- | --- | --- | --- | --- | --- |
| HELA | X | 11,12 | 12,13.3 | 8,12 | 9,10 | 16,18 | 7 | 8,12 | 9,10 |
| HK1 | X | 11,13 | 11 | 8,11 | 9,11 | 18,19 | 7,9 | 8,11 | 11,12 |
| C666-1 | XY | 11 | 8,11 | 11,12 | 10 | 17,18 | 6,8 | 8,11 | 11,16 |

Notes: HK1 and C666-1 were not contaminated to Hela cells.

**Supplementary table 2. Cytokine array of supernatants from the macrophages treated with rISG15. Upregulated cytokines more than 1.5 times are presented.**

| Cytokine | ISG15 0nM | ISG15 59nM | ISG15 295nM | Ratio of 59 nM/0nM |
| --- | --- | --- | --- | --- |
| PARC | 490 | 2,873 | 5,028 | 5.863265306 |
| Eotaxin | 163 | 645 | 870 | 3.957055215 |
| BTC | 814 | 2,926 | 1,375 | 3.594594595 |
| I-309 | 566 | 1,936 | 1,690 | 3.42351901 |
| IL-4 | 483 | 1,379 | 2,485 | 2.854037267 |
| IGF-I | 3,429 | 9,664 | 9,457 | 2.818168562 |
| HGF | 972 | 2,489 | 327 | 2.560699588 |
| HCC-4 | 3,815 | 9,571 | 6,289 | 2.508781127 |
| axl | 3,374 | 8,089 | 5,034 | 2.397302905 |
| GRO-alpha | 3,427 | 7,908 | 4,166 | 2.307557631 |
| PDGF-BB | 4,824 | 10,570 | 8,357 | 2.191127695 |
| IL-7 | 163 | 332 | 486 | 2.036809816 |
| SCF | 3,802 | 7,584 | 9,102 | 1.994739611 |
| Eotaxin-2 | 3,352 | 6,480 | 4,407 | 1.933174224 |
| IGFBP-4 | 1,220 | 2,337 | 3,704 | 1.915949159 |
| NT-3 | 3,799 | 6,993 | 8,186 | 1.840747565 |
| IL17 | 2,620 | 4,803 | 3,115 | 1.833556022 |
| BMP-6 | 966 | 1,691 | 1,210 | 1.75 |
| GM-CSF | 485 | 813 | 805 | 1.67628866 |
| MCP-2 | 5,544 | 8,775 | 9,421 | 1.582792208 |
| GRO | 5,284 | 8,285 | 5,705 | 1.568089335 |
| MDC | 5,940 | 9,308 | 8,319 | 1.567003367 |
| MIP-1-delta | 1,422 | 2,222 | 2,082 | 1.562587904 |

**Supplementary Figure Legends**

**Supplementary figure 1. A**, Activation of SFK signaling by phosphorylation of Tyr416 in a time-dependent manner in ISG15-induced macrophages. **B**, The presence of LFA-1 inhibitor, A286982, abolished the enhanced migration of NPC cells cocultured with CM of ISG15-induced macrophages.

**Supplementary figure 2.** The association between the number of ISG15^+^ CD163^+^ TAMs and clinical features in NPC biopsy.

**Supplementary figure 3.** Representative flow cytometric analysis of IFN-γ^+^, perforin^+^, and Granzyme B^+^ cells within CD8^+^ T cells in vitro cocultured with the conditional CM from ISG15-induced macrophages over time.
