# Supplementary figures and images for: Tumor Cell-Secreted ISG15 Promotes Tumor Cell Migration and Immune Suppression by Inducing the Macrophage M2-Like Phenotype
Source: Front Immunol. 2020 Dec 23;11:594775. doi: 10.3389/fimmu.2020.594775 (PMC7785797; doi:10.3389/fimmu.2020.594775)

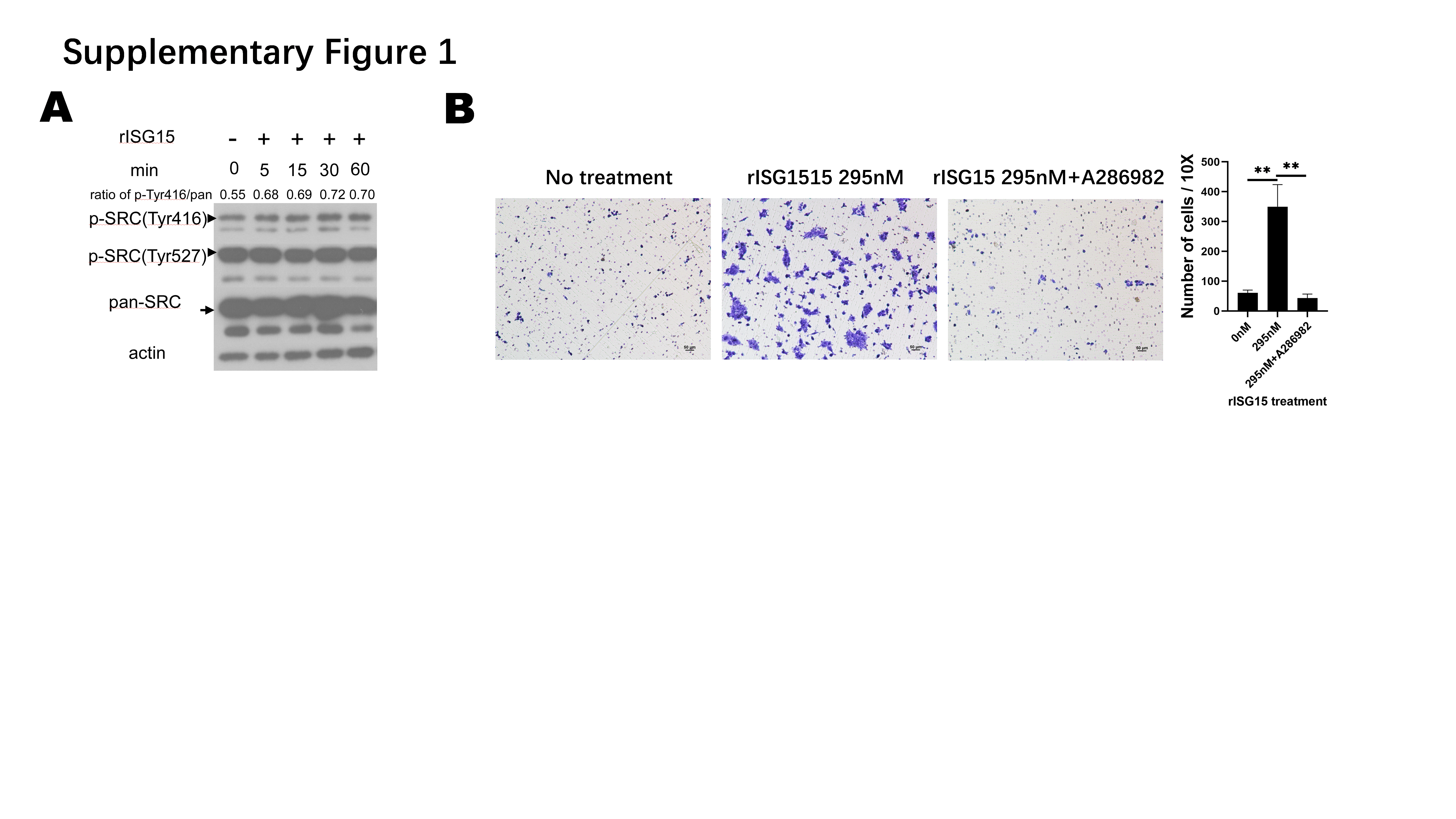

Supplement: Supplementary file 2 [file Image_1.tif]

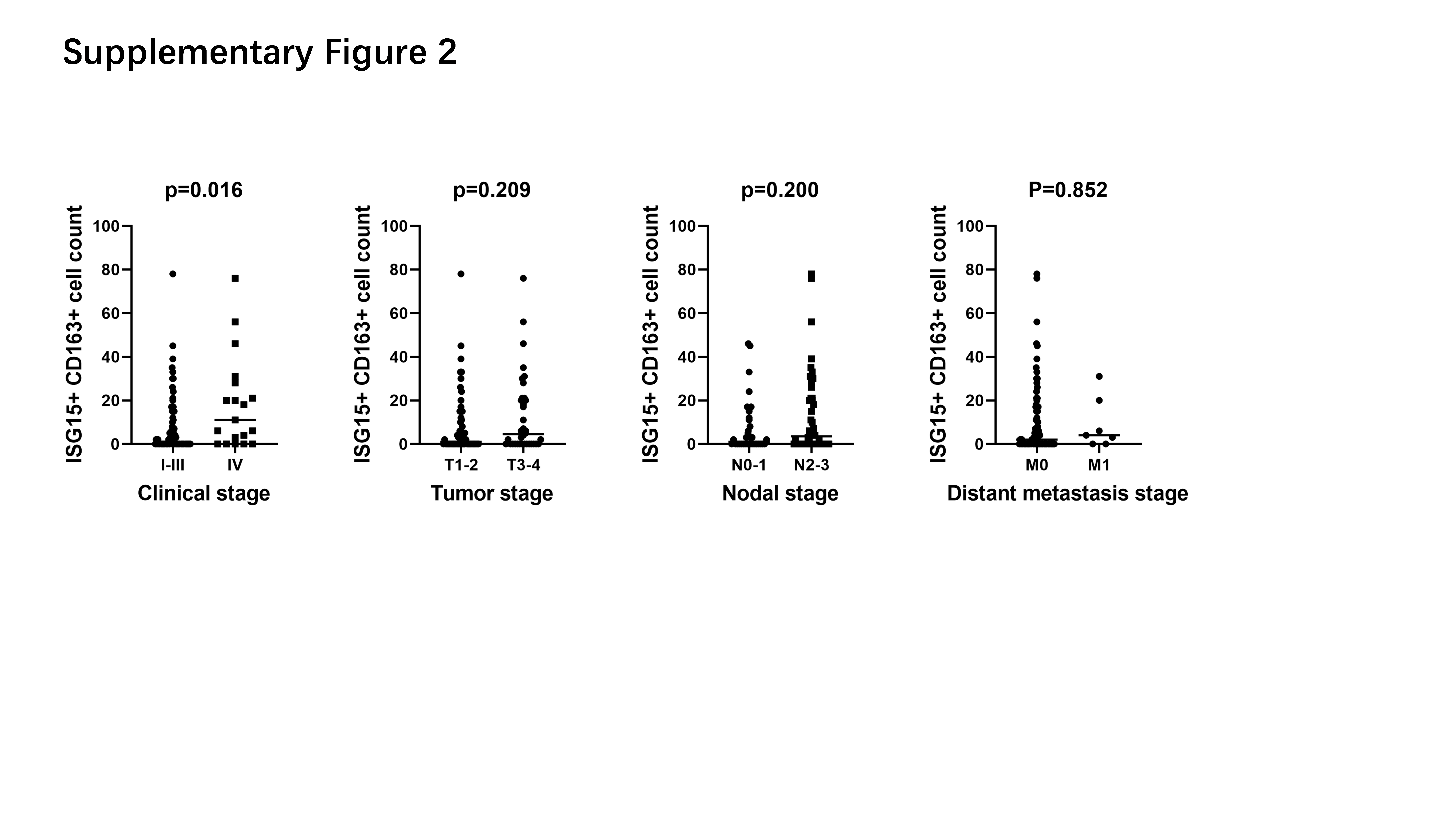

Supplement: Supplementary file 3 [file Image_2.tif]

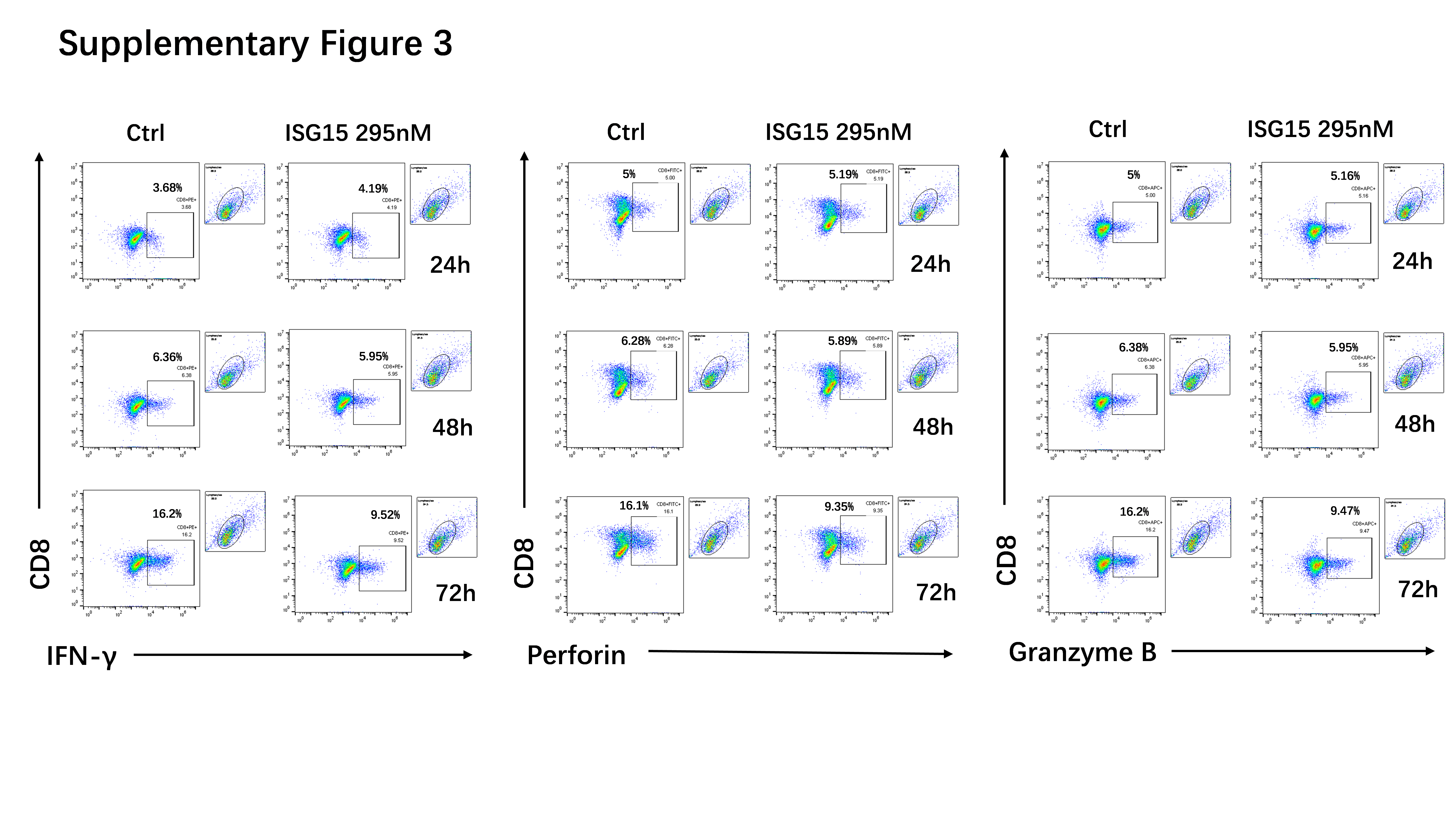

Supplement: Supplementary file 4 [file Image_3.tif]
